# Supplementary material for: The Anion Channel TMEM16a/Ano1 Modulates CFTR Activity, but Does Not Function as an Apical Anion Channel in Colonic Epithelium from Cystic Fibrosis Patients and Healthy Individuals
Source: Int J Mol Sci. 2023 Sep 18;24(18):14214. doi: 10.3390/ijms241814214 (PMC10531629; doi:10.3390/ijms241814214)

Supplementary material and figures

Table S1: Name, brief description and sequence of the primers for RT-qPCR of the genes which were analyzed in this study.

Gene	Product; Description	Primer sequence for RT-qPCR
ACTB	actin beta; reference gene for RT-qPCR	5´-CGAGGACTTTGATTGCACATTGTT; 5´-TGGGGTGGCTTTTAGGATGG
ALPI	intestinal alkaline phosphatase	5´-CGCTTTAACCAGTGCAACAC; 5´-GTCACCACTCCTACTGACTTTC
CFTR	CF transmembrane conductance regulator	5´-TTAATGCCCTTCGGCGATGT ; 5´-ATCGCGATAGAGCGTTCCTC
CLDN1	claudin 1; tight junction protein	5´-CTGGGAGGTGCCCTACTTTG; 5´-ACACGTAGTCTTTCCCGCTG
CLDN2	claudin 2; tight junction protein	5´-GATCCTACGGGACTTCTACTCA; 5´-CAGGGAGAACAGGGAAGAAATAA
CLDN3	claudin 3; tight junction protein	5´-ACGCGAGAAGAAGTACACGG; 5´-TAGACGTAGTCCTTGCGGTC
CLDN4	claudin 4; tight junction protein	5´-TTCTACAATCCGCTGGTGGC; 5´-GCGGAGTAAGGCTTGTCTGT
CLDN7	claudin 7; tight junction protein	5´-GGGGGAGACGACAAAGTGAA; 5´-CATACCAGGAGCAAGCTACCA
CLDN8	claudin 8; tight junction protein	5´-TGCCCAAAAACGTGAGCTTG; 5´-TGTGCGATGGGAAGGTATCG
ENaC	epithelial sodium channel	5´-GGACTGCTTCTACCAGACATAC; 5´-TCGACAGGATGTTGATGTAGTG
GAPDH	glyceraldehyde-3-phosphate dehydrogenase; reference gene for RT-qPCR	5´-TGCACCACCAACTGCTTAGC; 5´-GGCATGGACTGTGGTCATGAG
Ki-67	antigen Ki-67; proliferating cell marker	5´-GACCTCAAACTGGCTCCTAATC; 5´-GCTGCCAGATAGAGTCAGAAAG
LGR5	leucine rich repeat containing G protein-coupled receptor 5; intestinal stem cell marker	5´-GGGAAACGCTCTGACATACA; 5´-CTTCTGTGGGTACGTGTCTTAG
MUC2	mucin 2; goblet cell marker	5´-AGTTTGGGGAGCACTTCGAG; 5´-TCTTCCACGCAGTGGGTAAC
NHE3	Na^{+}/H^{+} exchanger 3 (SLC9A3)	5´-ACCGTGCGCTACACCATGAAGATG; 5´-ATGCGGTAGCGGTTCAGAAGCC
NKCC1	Na^{+}-K^{+}-$2Cl^{-}$ cotransporter 1 (SLC12A2)	5´-AAAGGAACATTCAAGCACAGC; 5´-CTAGACACAGCACCTTTTCGTG
OCLN	occludin; tight junction protein	5´-TCGACCAATGCTCTCTCAGC; 5´-CTCCTGGAGGAGAGGTCCAT
SLC26A3	solute carrier family 26 member 3; Cl^{-}/HCO_3^{-} exchanger	5´-CCAGCGTCTATTCCCTCAAAT; 5´-TCCCAGCAAATCCTCTGAATAC
TMEM16a	transmembrane protein 16a	5´-TTCCCAGCTACCTCACTAA; 5´-GGCCATGGAGATTCTGTAGATG
TMEM16f	transmembrane protein 16f	5´-AAGCAGCCCTTGGATCTTATC; 5´-GAAAGCAAGCCACTCCTACA
WDR43	WD repeat domain 43; crypt transit amplifying cell marker	5´-ATGGCACTTGGTTTCAGCCTA; 5´-ATCACTGGTGTCCTCACCTTT
ZO-1	Zonula occludens-1; tight junction protein	5´-CCTGAGTTTGACAGTGGAGTT; 5´-GCTGAAGGACTCACAGGAATAG

Table S2: Characteristics of human subjects that donated biopsies for establishing organoid cultures.

Sample	Age (years)	Gender	Biopsy section	Genotype
HL-1	35	Male	Transverse Colon	Healthy
HL-2	19	Female	Rectum	Healthy
CF-3	17	Female	Rectum	F508del/N1303K
CF-4	17	Male	Rectum	F508del/G542X
CF5	15	Female	Rectum	F508del/CFTRdel2,3(21kb)
CF7	14	Male	Rectum	F508del/ 2184delA
CF8	14	Male	Rectum	F508del/E822X
CF9	14	Female	Rectum	F508del/CFTRdel2,3(21kb)
CF10	14	Female	Rectum	F508del/ F508del
CF11	14	Male	Rectum	F508del/ F508del
CF12	45	Male	Rectum	F508del/R553X
CF14	20	Female	Rectum	F508del/G542X
CF15	32	Male	Rectum	F508del/ F508del
CF16	15	Female	Rectum	F508del/1078delT
CF17	19	Male	Rectum	F508del/ F508del
CF18	16	Male	Rectum	F508del/ F508del
CF19	24	Female	Rectum	F508del/ F508del
CF20	14	Female	Rectum	F508del/ F508del
CF21	44	Male	Rectum	F508del/ F508del
CF22	41	Female	Rectum	F508del/ F508del

Supplementary method

Human organoid cultures

Organoid were maintained in expansion medium (EM) which was composed of advanced DMEM F12 medium supplemented with 50% (v/v) L-WRN conditioned medium, 100 U penicillin/streptomycin, 10 mM HEPES pH 7.4, 0.2 mM GlutaMAX , 1x B27 supplement , 1x N2 supplement , 1 mM N-acetylcysteine, 50 ng/mL human EGF, 1 µg/mL [Leu-15]gastrin, 500 nM A83-01, 10 µM SB202190, 100 µg/mL Primocin and 10 nM Prostaglandin E2 for a period of 6-7 days. EM was supplemented with 10 µM of Y-27632 and 10 µM of CHIR-99021 during the initial 2 days of the expansion. Organoids were passaged every 6-8 days after mechanical dissociation in a cold advanced DMEM F12 medium.

To prepare differentiation medium (DM), L-WRN conditioned medium, SB202190, A83-01 and Prostaglandin E2 were excluded and 15% (v/v) of murine Noggin conditioned medium were included in the EM composition. Sources for growth medium components and small molecules are listed in table S3.

Table S3: Sources for growth medium components and small molecules used for organoid culture

Material	Source
Cultrex	Bio-Techne, Wiesbaden, Germany
Matrigel	Corning, Kaiserslautern, Germany
Jagged-1	ANASPEC, Fremont, CA, USA
CHIR-99021	Sigma-Aldrich, Darmstadt, Germany
PGE2	Sigma-Aldrich, Darmstadt, Germany
Nicotinamid	Sigma-Aldrich, Darmstadt, Germany
EMEM	Lonza, Cologne, Germany
non-essential amino acids	Gibco, Darmstadt, Germany
penicillin/streptomycin	Sigma-Aldrich, Darmstadt, Germany
collagen IV	Sigma-Aldrich, Darmstadt, Germany
advanced DMEM F12 medium	Gibco, Darmstadt, Germany
Trypl E	Gibco, Darmstadt, Germany
Forskolin	Sigma-Aldrich, Darmstadt, Germany
3-Isobutyl-1-methylxanthine 1 g (IBMX)	Sigma-Aldrich, Darmstadt, Germany
Ani9	Sigma-Aldrich, Darmstadt, Germany
Ani9	Tocris Bioscience, Minneapolis, MN, USA
SB 202190	Tocris Bioscience, Minneapolis, MN, USA
EGF recombinant human protein	Peprotech, Hamburg, Germany
Advanced DMEM/F12	Gibco, Darmstadt, Germany
Y-27632 RhoA/ ROCK inhibitor	Tocris Bioscience, Minneapolis, MN, USA
Primocin	Invivogen, Darmstadt, Germany
HEPES	Gibco, Darmstadt, Germany
GlutaMAX	Gibco, Darmstadt, Germany
B27 supplement	Gibco, Darmstadt, Germany
N2 supplement	Gibco, Darmstadt, Germany
Gastrin	Sigma-Aldrich, Darmstadt, Germany
TrypLE Express	Gibco, Darmstadt, Germany
Cell Recovery Solution	Corning, Kaiserslautern, Germany
N-acetylcysteine	Sigma-Aldrich, Darmstadt, Germany
EDTA	PanReac AppliChem, Darmstadt, Germany

Supplementary Figures

Figure S1: transepithelial voltage and resistance for CM-CE coculture (A) and differentiated (B) colonoid monolayers treated with TMEM16a-specific inhibitor Ani9 (30μM, luminal; Red trace) or DMSO vehicle control (Black trace), corresponding to the Ieq data presented in figure 3.

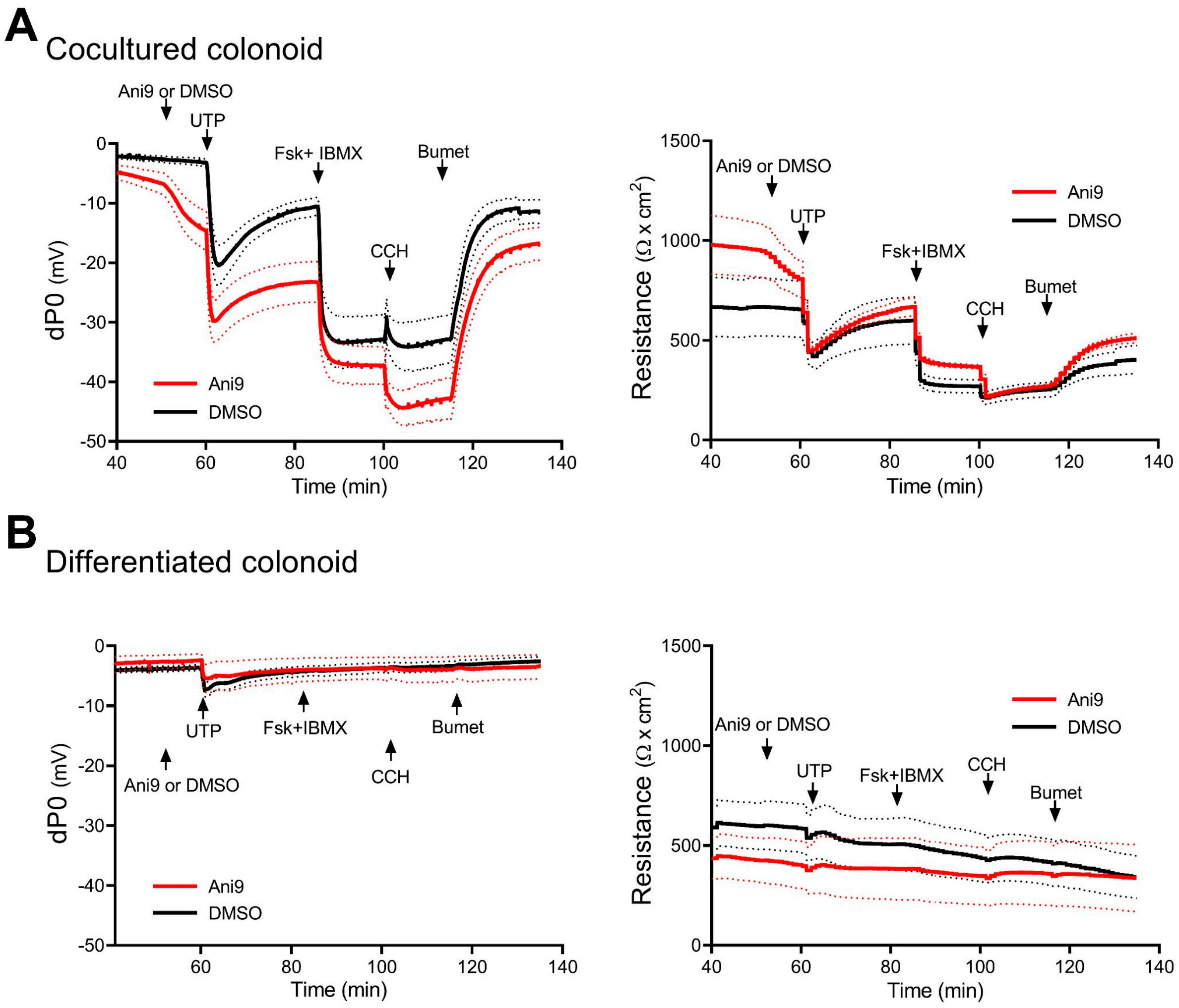

Figure S2: multiple sequence alignment for canonical mouse Tmem16a protein sequence (Q8BHY3-1=canonical) and four reported isoforms of human TMEM16a (Q5XXA6-1=canonical, Q5XXA6-2, Q5XXA6-4, Q5XXA6-5) using PRALINE multiple sequence alignment toolbox. The figure only shows regions of the alignment, which correspond to the epitopes sequences (mouse protein) used to generate Ano1-132, Ano1-133 and Ano1-134 antibodies. Ano1-132 and Ano1-133 epitopes are mostly conserved, particularly between mouse and human canonical TMEM16a sequences, and Ano1-134 epitope is completely conserved among all of the tested isoforms.

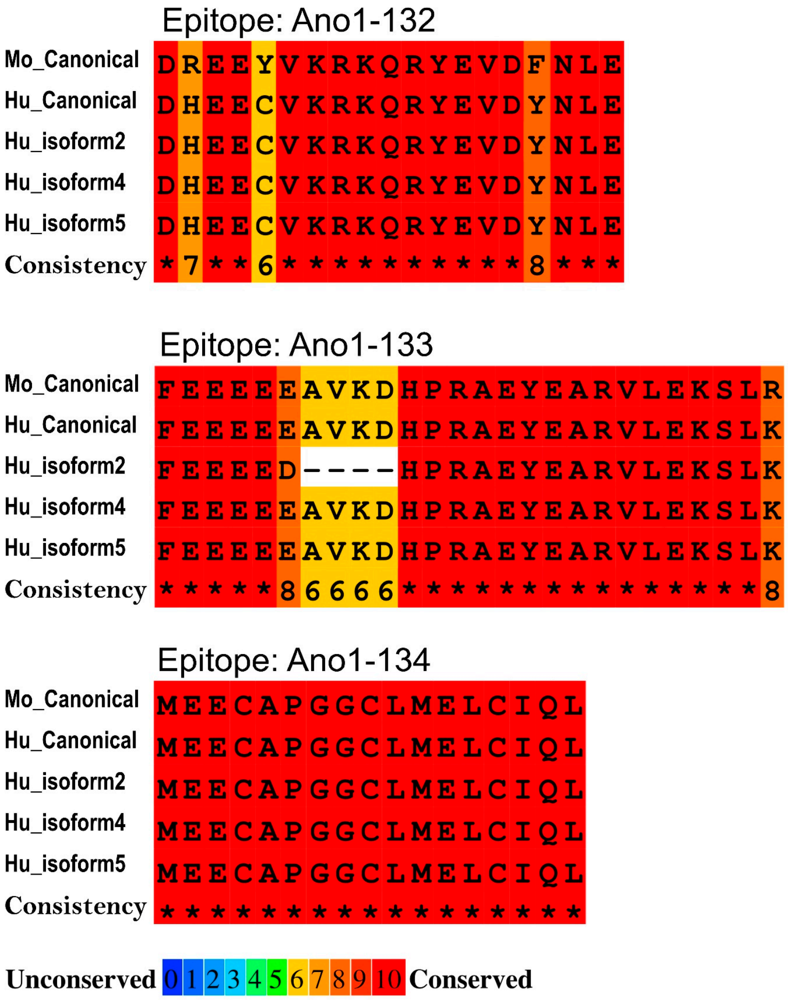

Figure S3: Transepithelial voltage and resistance of isolated intestinal mucosa from A) jejunum (wt n=9, mt n=6), B) ileum (wt n=8, mt n=5), C) proximal colon (wt n=9, mt n=4) and D) mid-distal colon (wt n=11, mt n=10) of homozygous F508del mice (mt, red trace) and wild type littermates (wt, black trace), corresponding to Ieq data presented in figure 5.

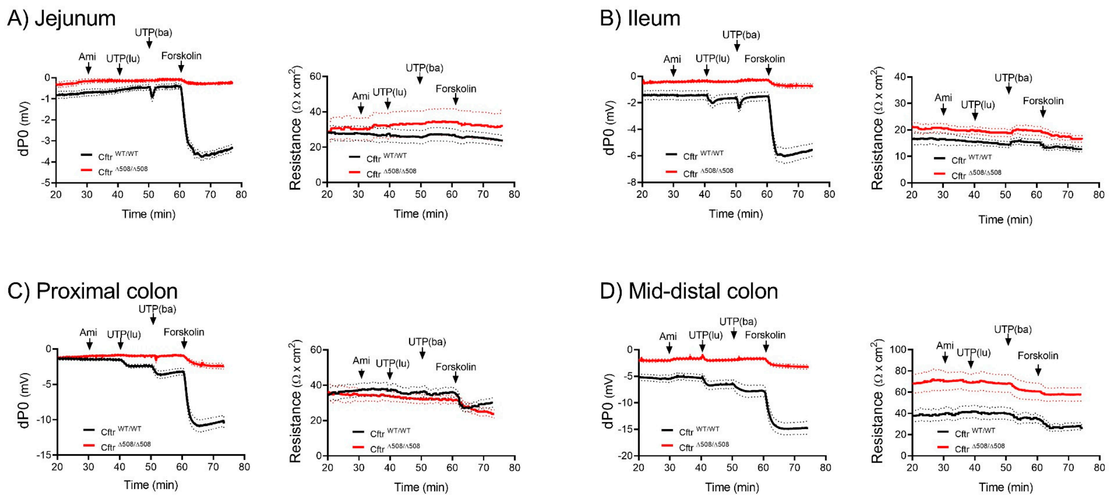

Figure S4: transepithelial voltage and resistance for RM-RE cocultured monolayers treated with TMEM16a-specific inhibitor Ani9 (30µM, luminal; Red trace) or DMSO vehicle control (Black trace), corresponding to the Ieq data presented in figure 7.

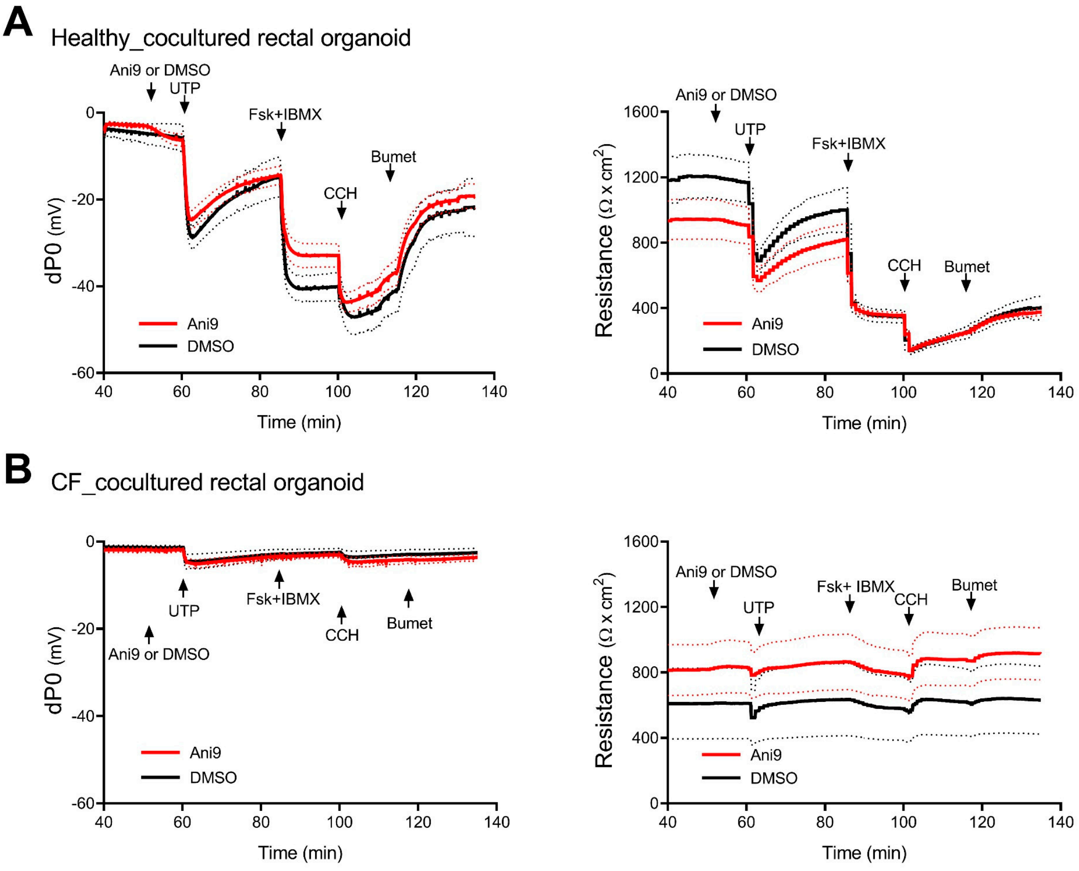

Figure S5: comparison of RM-RE cocultures established from healthy (HL) and CF subjects for expression of a panel of marker genes for proliferation, ion transport, differentiation and barrier function using RT-qPCR.

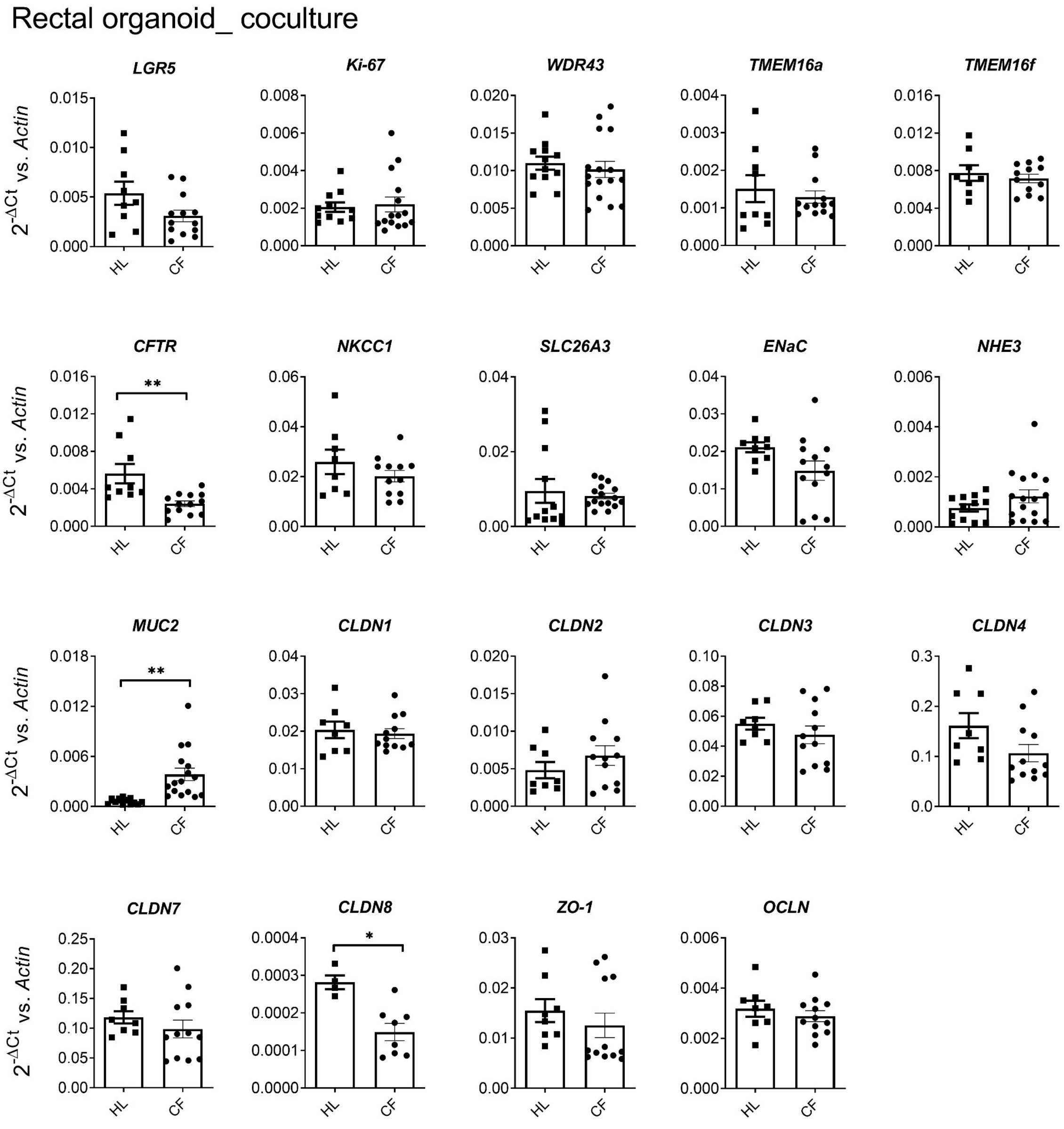

Figure S6: transepithelial voltage and resistance for differentiated rectal organoid monolayers treated with TMEM16a-specific inhibitor Ani9 (30µM, luminal; Red trace) or DMSO vehicle control (Black trace), corresponding to the Ieq data presented in figure 8.

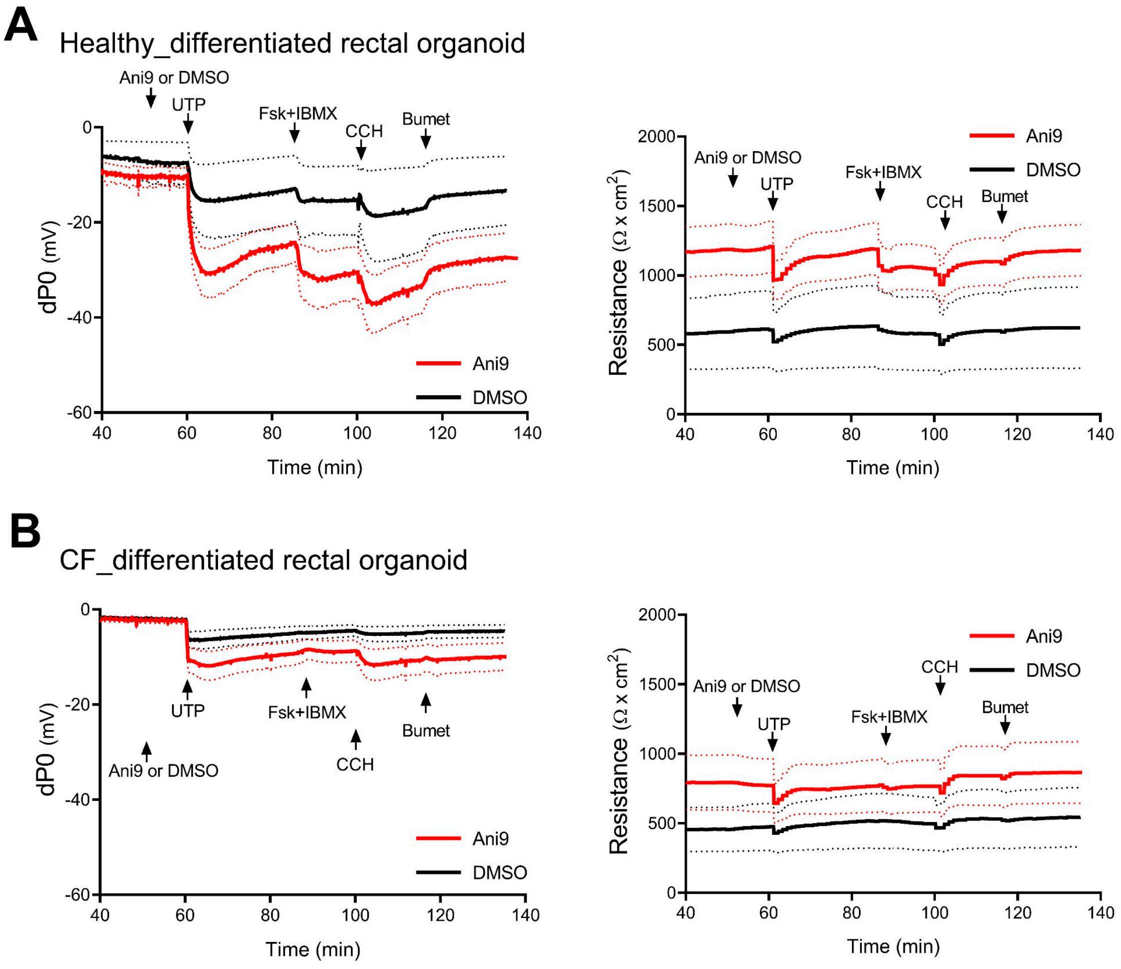

Supplement: Supplementary file 1 [file ijms-24-14214-s001.zip › ijms-2585266-supplementary.pdf]
