# Supplementary figures and images for: The Anion Channel TMEM16a/Ano1 Modulates CFTR Activity, but Does Not Function as an Apical Anion Channel in Colonic Epithelium from Cystic Fibrosis Patients and Healthy Individuals
Source: Int J Mol Sci. 2023 Sep 18;24(18):14214. doi: 10.3390/ijms241814214 (PMC10531629; doi:10.3390/ijms241814214)

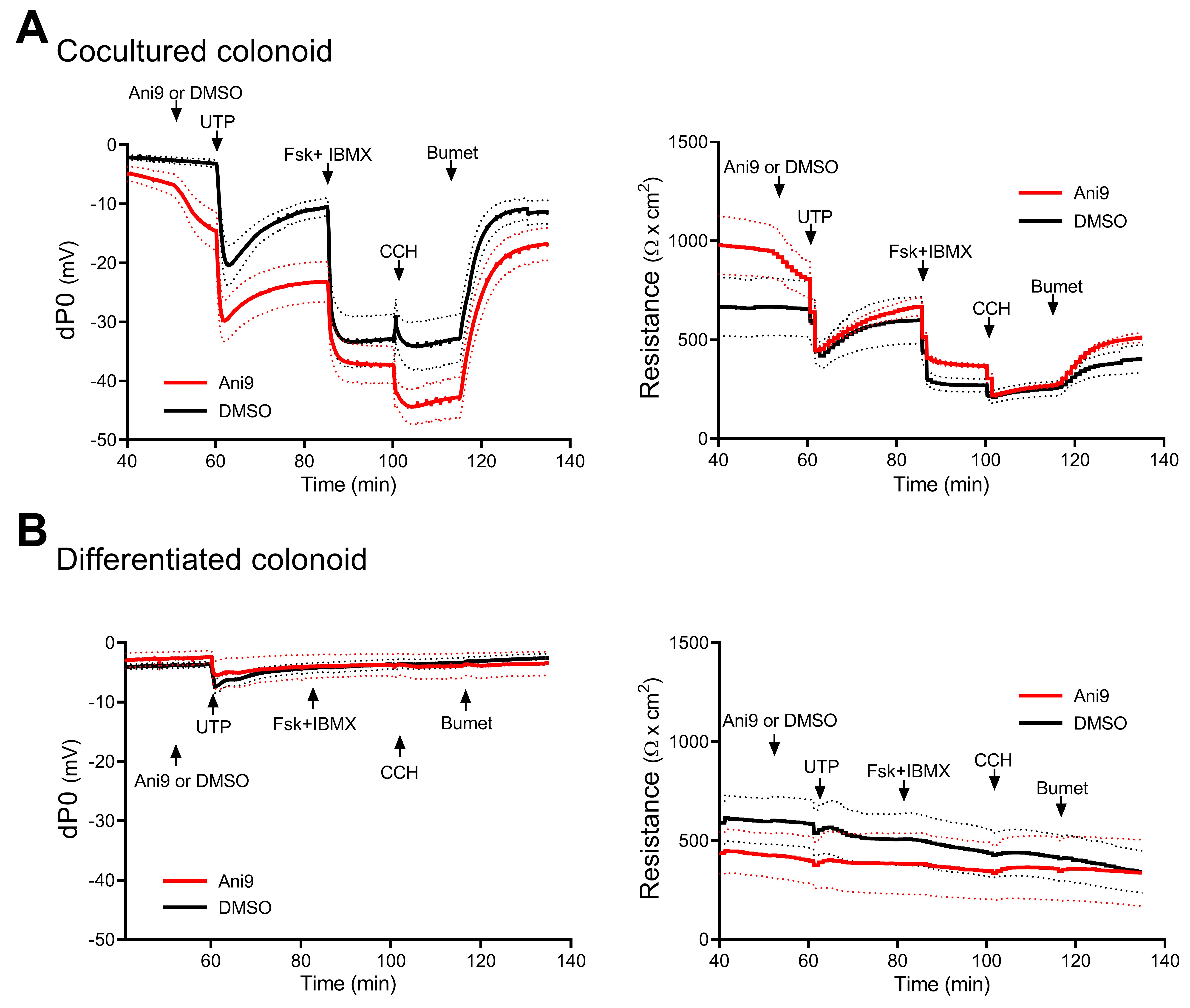

Supplement: Supplementary file 1 [file ijms-24-14214-s001.zip › Figure S1.jpg]

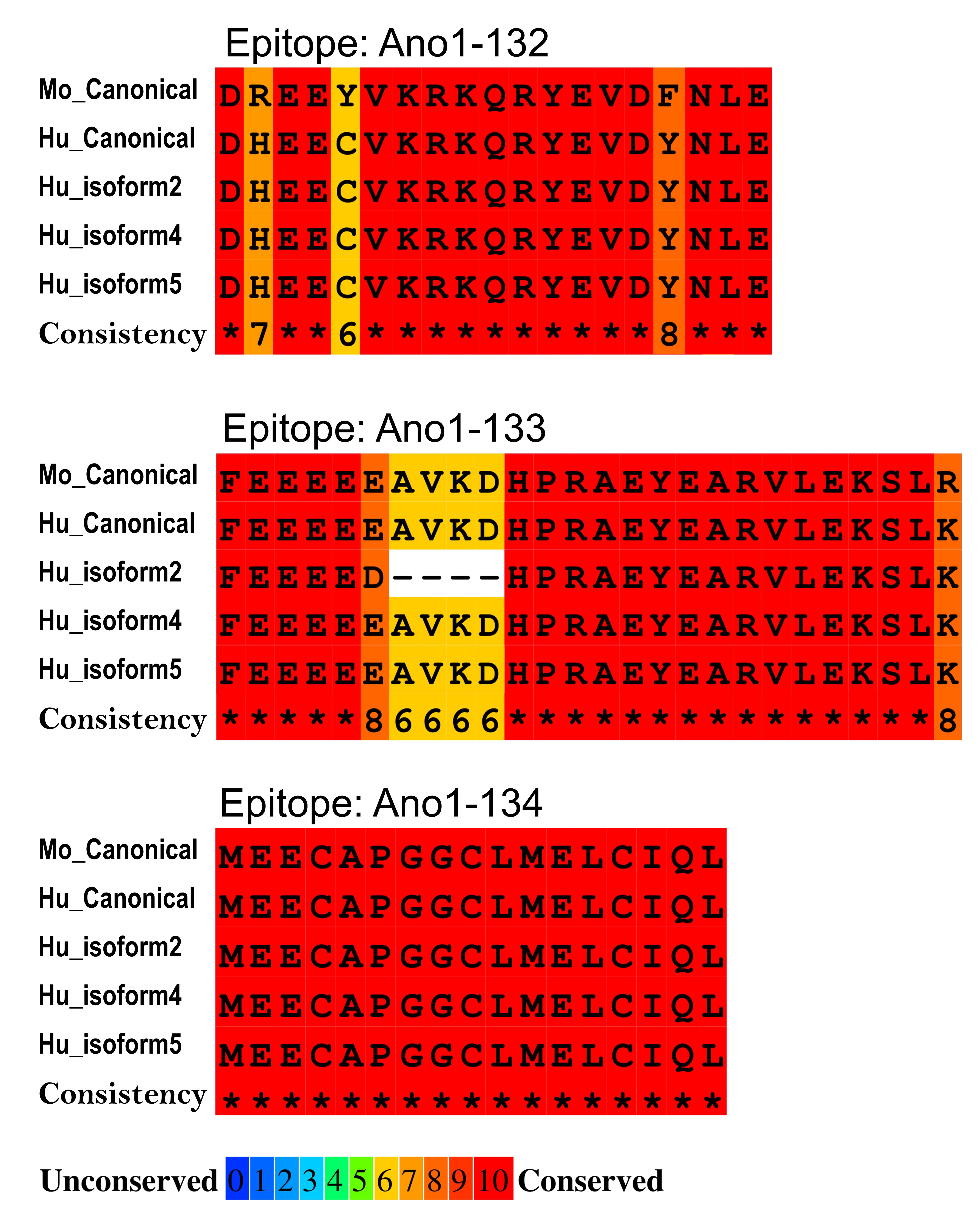

Supplement: Supplementary file 1 [file ijms-24-14214-s001.zip › Figure S2.jpg]

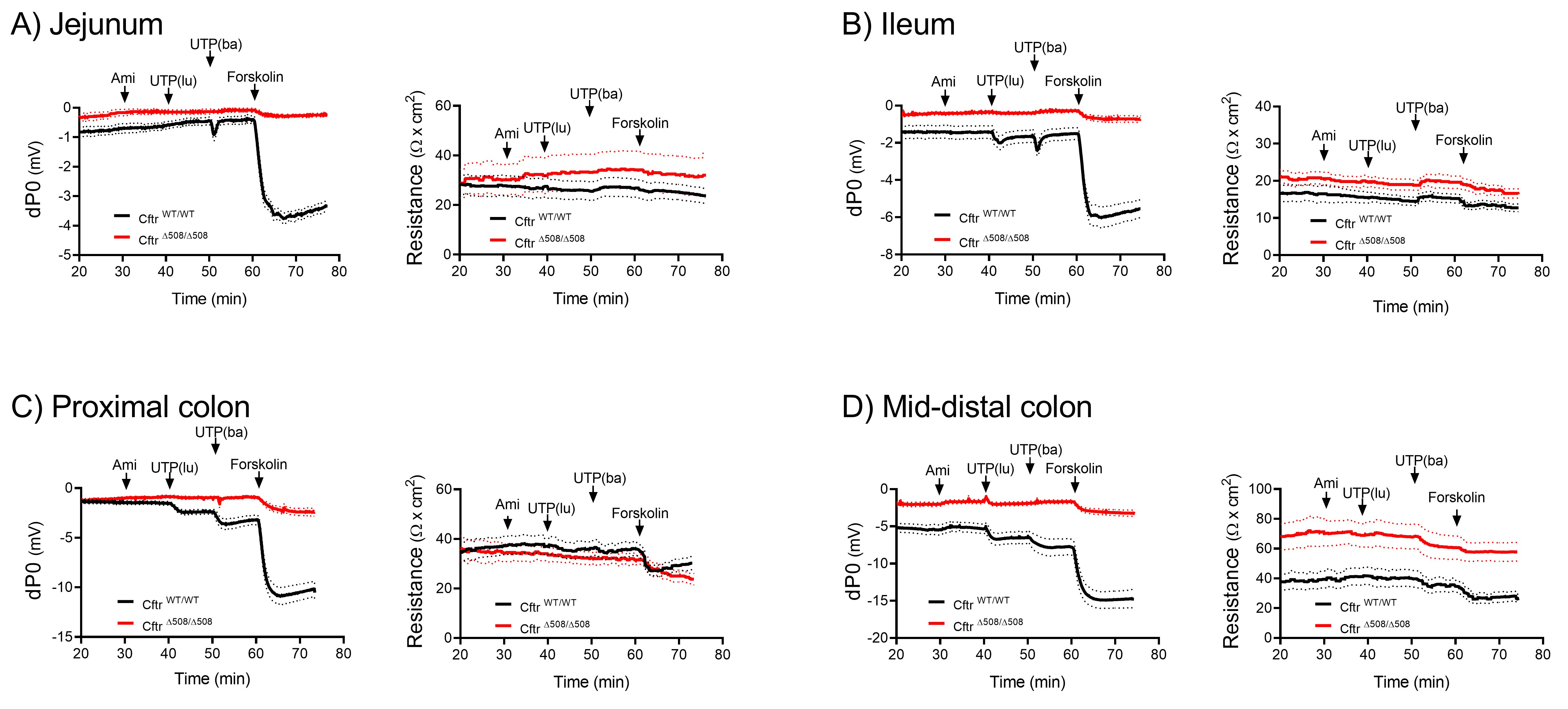

Supplement: Supplementary file 1 [file ijms-24-14214-s001.zip › Figure S3.jpg]

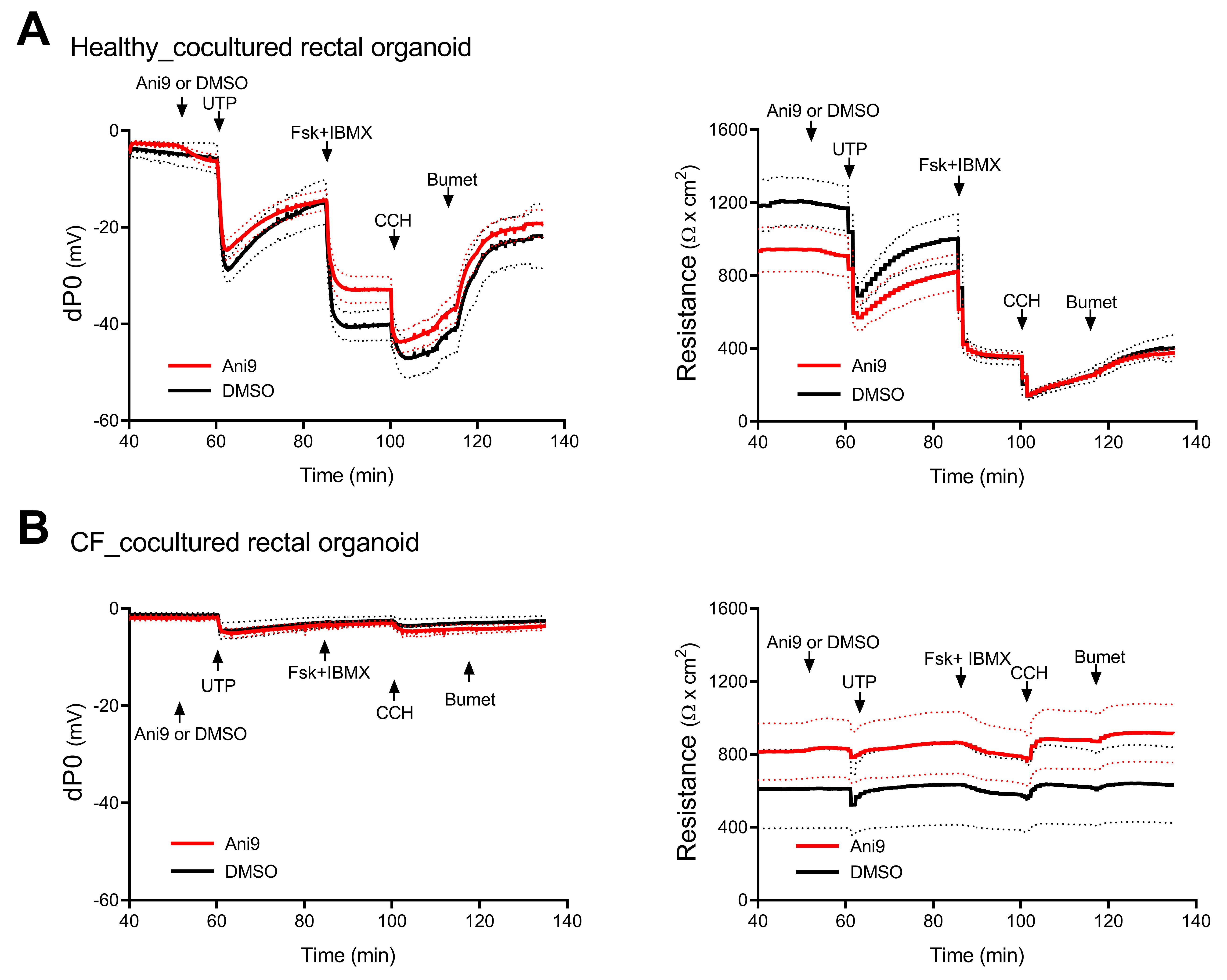

Supplement: Supplementary file 1 [file ijms-24-14214-s001.zip › Figure S4.jpg]

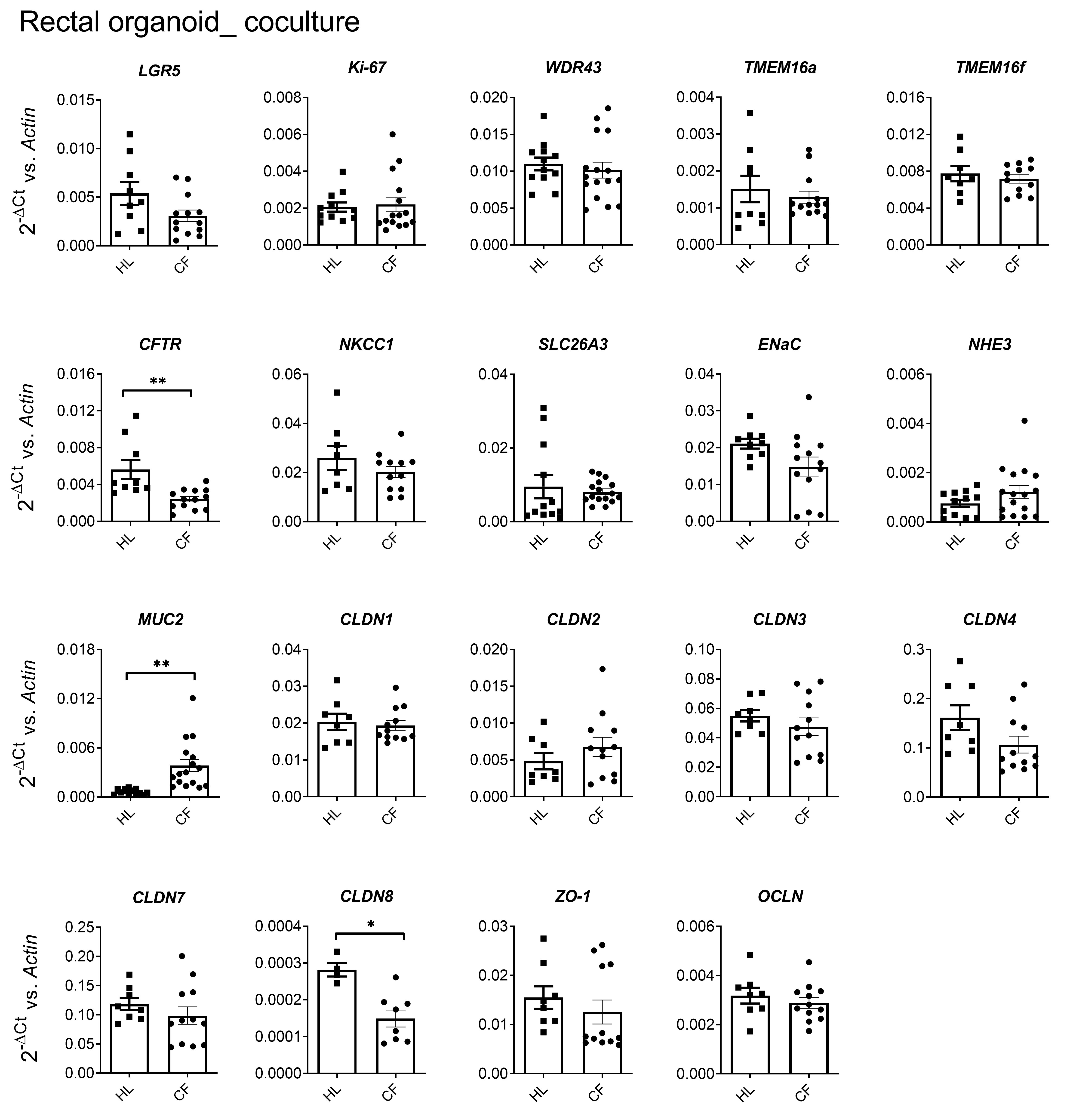

Supplement: Supplementary file 1 [file ijms-24-14214-s001.zip › Figure S5.jpg]

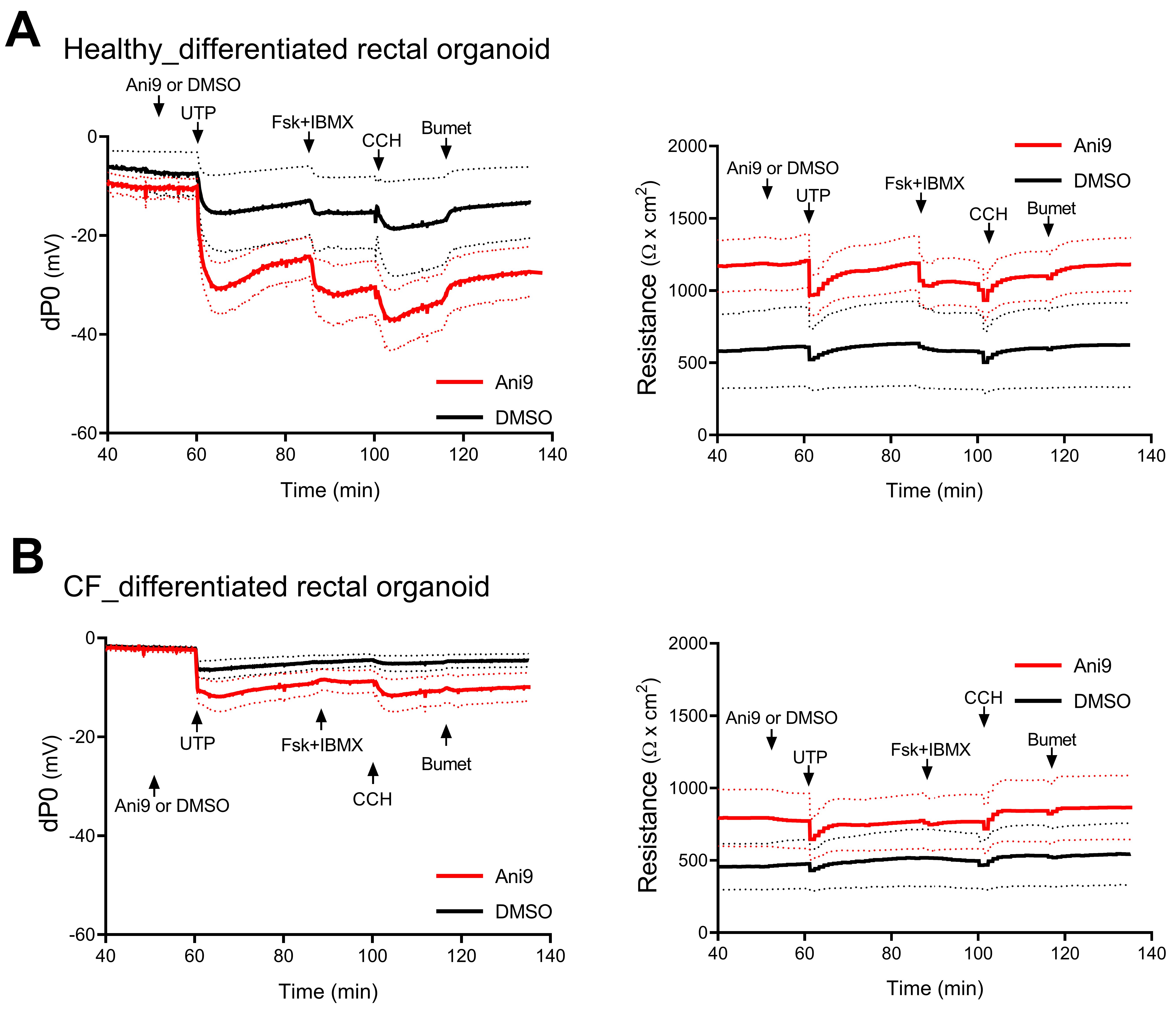

Supplement: Supplementary file 1 [file ijms-24-14214-s001.zip › Figure S6.jpg]
